# A novel endoscopy-guided airway management strategy for lateral decubitus endoscopic procedures

**DOI:** 10.3389/fmed.2026.1934908

**Published:** 2026-10-13

**Authors:** Changlong Qiao, Jing Zhou, Meng Lv, Lei Guo

**Affiliations:** 1 Department of Anesthesiology, The First Affiliated Hospital of Shandong First Medical University & Shandong Provincial Qianfoshan Hospital, Shandong Institute of Anesthesia and Respiratory Critical Medicine, Shandong Provincial Clinical Research Center for Anesthesiology, Jinan, China; 2 Department of Obstetrics and Gynecology, The First Affiliated Hospital of Shandong First Medical University & Shandong Provincial Qianfoshan Hospital, Laboratory of Laparoscopic Technology, the First Affiliated Hospital of Shandong First Medical University, Jinan, China; 3 Department of Anesthesiology, Shandong Provincial Hospital of Shandong First Medical University, Jinan, Shandong, China

**Keywords:** airway management, difficult airway, endotracheal intubation, gastrointestinal endoscopy, hemodynamic stability, lateral decubitus

## Abstract

**Background:**

This study investigates the feasibility and safety of gastrointestinal endoscopy-guided endotracheal intubation in patients undergoing elective gastrointestinal endoscopic surgery under general anesthesia requiring airway management in the lateral decubitus position.

**Methods:**

Forty patients undergoing elective gastrointestinal endoscopy in the left lateral decubitus position were enrolled. Standardized anesthesia induction included midazolam, etomidate, sufentanil, and atracurium. Under endoscopic visualization, endotracheal intubation was performed using a stiff stylet-assisted tube inserted through a bite block's right lateral port. Primary outcomes were first-attempt success rate and intubation time. Secondary outcomes included hemodynamic stability, adverse events, and procedural complications.

**Results:**

The first-attempt intubation success rate was 100%. The mean intubation time was 30.95 ± 6.58 s. Hemodynamic parameters showed a transient hypotensive response following induction in 15 out of 40 patients (37.5%); MAP had returned to ≥65 mmHg by the post-intubation measurement, and no vasopressor agents were administered. Repeated-measures ANOVA revealed a statistically significant main effect of time on both MAP [F (3.048, 118.888) = 23.05, *p* < 0.001, partial *η*^2^ = 0.372] and HR [F (4, 156) = 3.258, *p* = 0.013, partial *η*^2^ = 0.077], with a transient stress response observed post-intubation (maximal increase: MAP +4.5%, HR +6.1% vs. baseline). No severe complications (e.g., esophageal intubation, dental injury, hypoxia) occurred (0/40; 95% CI: 0%–8.8%). Mild sore throat was observed in 22.5% of cases, resolving spontaneously within 24 h.

**Conclusion:**

In this feasibility cohort, gastrointestinal endoscopy-guided endotracheal intubation demonstrated a 100% first-attempt success rate with a mean intubation time of 30.95 ± 6.58 s and no severe complications. These preliminary findings suggest that the technique appears to be a feasible and safe option for airway management in patients undergoing lateral-position gastrointestinal endoscopy. However, these findings are from an uncontrolled, single-center study with a limited sample size. Larger comparative studies are warranted to confirm these results.

**Clinical Trial Registration:**

https://clinicaltrials.gov/, identifier NCT06990490.

## 1 Introduction

The supine position remains the standard posture for endotracheal intubation in operating rooms. This conventional approach facilitates airway management through optimal anatomical alignment and familiar ergonomics for anesthesiologists (1). Previous studies have revealed that intubation in the lateral position is more difficult and time-consuming compared to the supine position, due to the distortion of the airway and the operator might have to adopt a posture which they may not be accustomed (2, 3). However, endotracheal intubation performed in the lateral position may be advantageous for patients undergoing surgical procedures in the lateral position, because it avoids the risk of adverse events associated with transitioning an anesthetized patient from the supine to any other position, and reduces the required time and workforce (4).

During gastroscopy performed with propofol anesthesia, patients are in the lateral position, the glottis becomes easily visible just before the gastrointestinal endoscope enters the esophageal entrance. This anatomical view suggests a potential for airway management. Recent studies have explored the role of endoscopic visualization for airway management. Barakat et al. reported a randomized trial of endoscopist-facilitated intubation with an endotracheal tube backloaded onto an ultra-slim gastroscope in patients undergoing ERCP (5). A case report from our institution also described the use of a standard gastroscope with a tube passed through the lateral port of a bite block in a single emergency patient with life-threatening variceal bleeding (6). Although this technique has been the standard airway management approach in our department for many years, its safety and efficacy have not been formally evaluated in a prospective cohort. The present study, therefore, represents the first prospective evaluation of this established technique, assessing the feasibility and safety of standard gastrointestinal endoscopy-guided endotracheal intubation using a stiff stylet-assisted tube delivered through the lateral port of a bite block in patients undergoing elective gastrointestinal endoscopy in the left lateral decubitus position. This approach fundamentally differs from FOB-guided intubation by utilizing equipment ubiquitous in endoscopy suites and incorporating a stiff stylet to enhance tube delivery. Thus, it was hypothesized that this novel gastrointestinal endoscopy-guided endotracheal intubation technique is feasible, safe, and advantageous for patients in the lateral position.

## 2 Materials and methods

### 2.1 Study design

This was a prospective feasibility cohort study conducted in The First Affiliated Hospital of Shandong First Medical University & Shandong Provincial Qianfoshan Hospital between February 2025 and March 2025. Data collection was completed in March 2025; the subsequent period was dedicated to data analysis, figure and table preparation, and internal manuscript review prior to submission. The study was conducted in accordance with the Declaration of Helsinki and reported following the Strengthening the Reporting of Observational Studies in Epidemiology (STROBE) statement for observational studies. The study was approved by the Ethics Committee of The First Affiliated Hospital of Shandong First Medical University & Shandong Provincial Qianfoshan Hospital [Approval No: YXLL-KY-2025(028)] and was registered at ClinicalTrials.gov (Registration No: NCT06990490). The first participant was enrolled on February 1, 2025, and the study was registered retrospectively on May 10, 2025. Furthermore, all participants provided their written informed consent for study participation and for the publication of de-identified procedural images and video. No identifiable images or data are presented in this manuscript. The same skilled anesthesiologist and gastrointestinal endoscopist performed all endotracheal intubation with patients in the lateral position.

### 2.2 Patients

Between February 1 and March 31, 2025, 40 of 40 eligible patients (100%) were enrolled consecutively; the 40th patient was enrolled on March 5, 2025. The two-month period was predefined in the study protocol to allow sufficient time for recruitment. Upon reaching the target sample size of 40 on March 5, 2025, we ceased further recruitment, and the remaining study period was dedicated to data verification and preparation for analysis. A total of 40 patients scheduled for elective gastrointestinal endoscopy under general anesthesia requiring tracheal intubation in the left lateral decubitus position were screened. This technique is the standard airway management approach in our department for lateral-position endoscopic procedures; therefore, all patients who met the clinical indication were enrolled, and none were excluded. The only exclusion criteria were (1): refusal to provide informed consent, and (2) severely restricted mouth opening that would preclude safe passage of the bite block and gastrointestinal endoscope, as this technique inherently requires transoral endoscope insertion. This study was designed as a real-world feasibility study reflecting routine clinical practice. We did not restrict enrolment based on ASA status, age, or BMI, as the technique was applied to all patients requiring tracheal intubation for lateral-position endoscopic procedures. In fact, for patients with higher ASA grades or hemodynamic instability, avoiding post-induction repositioning may be particularly beneficial.

As this investigation was designed as a feasibility study, no formal *a priori* sample size calculation was performed. The sample size of 40 was chosen to provide a preliminary evaluation of the technique's success rate and safety profile.

### 2.3 General anesthesia and endotracheal intubation

All patients underwent standardized preoperative fasting protocols, including ≥8 h of solid food restriction and ≥4 h of clear liquid abstinence. No preoperative medications were administered. Prior to airway management, subjects were placed in the supine position with continuous monitoring of vital parameters: heart rate (HR), non-invasive blood pressure, pulse oximetry (SpO₂), and bispectral index (BIS). Subsequently, patients were instructed to assume the left lateral decubitus position. A pillow was utilized to support the head and neck, ensuring the preservation of the spine's natural alignment in the horizontal plane throughout the process of anesthesia induction and intubation. The gastrointestinal endoscopy system was calibrated and the endoscope lumen adequately lubricated.

An appropriately sized reinforced endotracheal tube (Henan Tuoren Medical Device Group Co., Ltd., Henan, China; Model: cuffed-PVC reinforced type, internal diameters ranging from 6.5 to 7.5 mm for female patients and 7.0 to 8.0 mm for male patients) was prepared, a stiff stylet (Uetech™ Zhejiang Uetech Medical Device Co., Ltd., Taizhou, China) was inserted into the lumen without exceeding the distal lateral port of the tube, and thorough lubrication was ensured. The reinforced tube was selected because the wire-reinforced wall maintains patency even when the tube is flexed or angled; and the tube can be bent upward and backward after fixation to avoid interfering with the endoscopist's operation, given that the anesthesia machine is positioned behind the patient's head in the endoscopy suite layout. A disposable bite block (Hongxing™, Yangzhou Hongyuan Medical Device Factory, Yangzhou, China; Device Registration No: Su Yang Xie Bei 20140099; Model C) was placed between the incisor teeth to maintain mouth opening prior to the induction of anesthesia. An Olympus GIF-Q260J gastrointestinal endoscope (Olympus Corporation, Tokyo, Japan), used with an Olympus CV-290 video processor, was calibrated and the endoscope lumen adequately lubricated.

Following standardized preoxygenation (100% FiO_2_ for 3 min), general anesthesia was induced using a sequential intravenous protocol: midazolam 0.03-0.05 mg/kg, etomidate 0.15-0.3 mg/kg, sufentanil 0.2-0.4 μg/kg, and atracurium 0.3-0.6 mg/kg. This anaesthetic regimen is the standard induction protocol in our institution for interventional gastrointestinal endoscopy requiring tracheal intubation and reflects routine clinical practice rather than a study-specific intervention. Upon induction, ventilation was provided with a face mask employing a two-handed approach, all while ensuring the patient's standardized left lateral decubitus position was maintained.

Following confirmation of adequate neuromuscular blockade, endotracheal intubation was performed. All intubations were performed by a single anesthesiologist (5 years of clinical experience, >100 endoscopic-guided lateral-position intubations) working in collaboration with a single gastroenterologist (8 years of endoscopy experience, >200 guided intubation procedures). Face mask ventilation was discontinued immediately before endoscope insertion; thus, the interval from cessation of ventilation to re-established ventilation corresponds closely to the intubation time defined in this study (from endoscope insertion to circuit connection). The gastroenterologist introduced the gastrointestinal endoscope transorally through the central channel of the bite block. Meanwhile, the anesthesiologist positioned the endotracheal tube (preloaded with a stiff stylet) within the right lateral port of the bite block. To optimize glottic exposure and prevent tongue base collapse, the anesthesiologist used the left hand to perform a jaw thrust maneuver, while advancing the tube with the right hand under direct endoscopic visualization. Continuous suctioning of oropharyngeal secretions was maintained throughout endoscopic advancement. Once the glottis was clearly visualized, the tracheal tube was advanced from the lateral port and passed through the glottis into the trachea. The stylet was then removed, the tube depth adjusted, the cuff inflated, and the breathing circuit connected (Figure 1).

**Figure 1:**
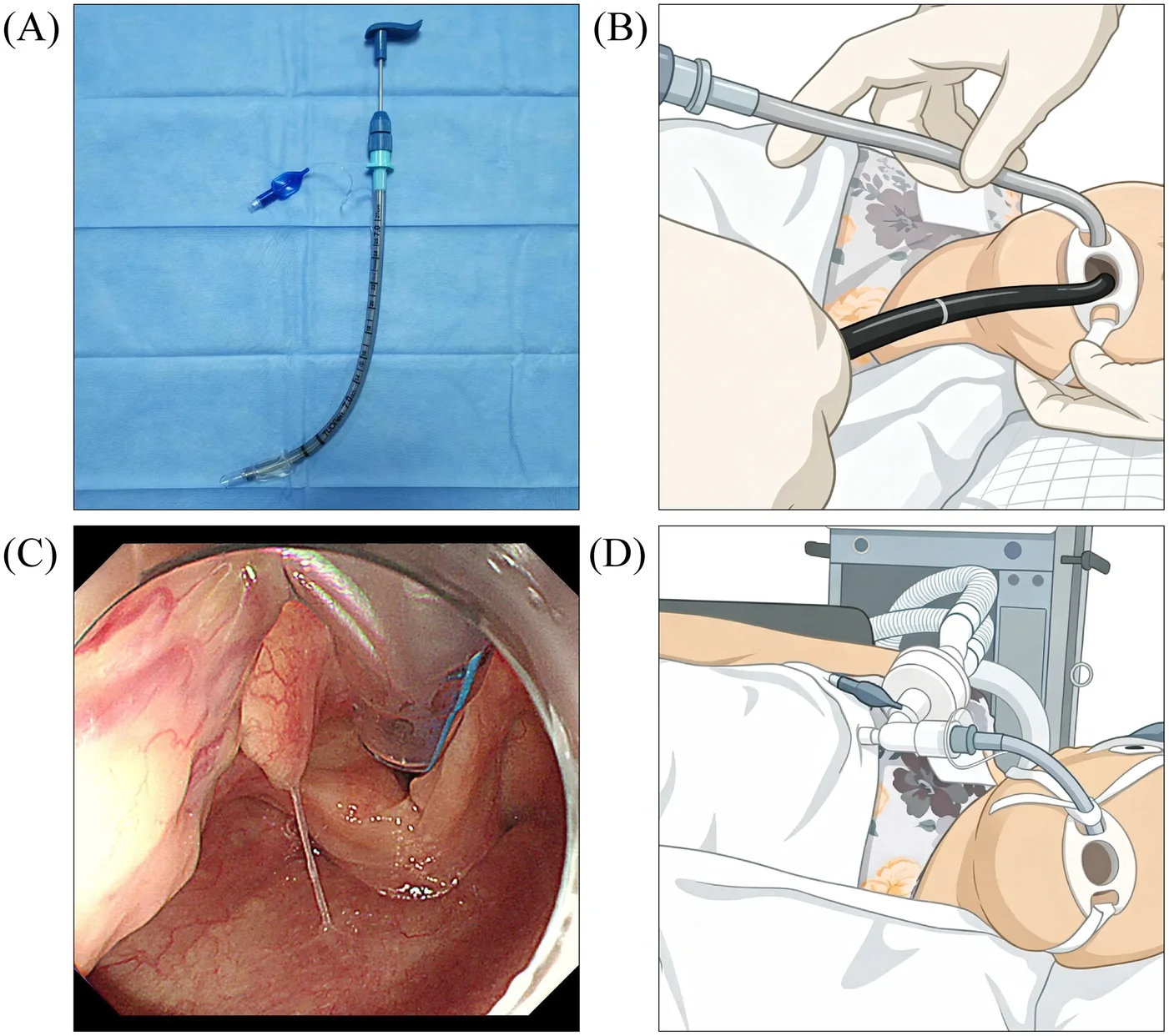
Schematic demonstration nof gastrointestinal endoscopy-guided endotracheal intubation in patients placed in the left lateral decubitus position. **(A)** Reinforced endotracheal tube preloaded with a stiff stylet. **(B)** Concurrent insertion of the gastrointestinal endoscope through the central channel and the tracheal tube via the right lateral port of the disposable bite block. **(C)** Real-time magnified endoscopic visualization of the glottis during intubation manipulation. **(D)** Final secure fixation of the endotracheal tube after successful tracheal cannulation. A Supplementary Video demonstrating the complete intubation maneuver is available as online Supplementary Material.

Intubation failure was defined *a priori* as intubation time exceeding 2 min or more than 2 intubation attempts. A prespecified rescue plan was established for failed intubation: (1) immediate deployment of the difficult-airway trolley, (2) summoning of a second experienced anesthesiologist, and (3) if the above measures failed, immediate return of the patient to the supine position for conventional laryngoscopic or video-laryngoscopic intubation. This rescue protocol was not required for any patient in this series.

Tracheal tube cuff pressure was maintained within the recommended range of 20–30 cmH₂O using a handheld cuff pressure manometer, consistent with our institutional standard practice. Cuff pressure was measured immediately after inflation and re-checked following any patient position change.

Tracheal tube placement was confirmed by waveform capnography, in addition to direct endoscopic visualization of the tube passing through the vocal cords.

Ventilation was initiated with tidal volume 6–8 mL/kg, respiratory rate 10–15 breaths/min, inspiratory-to-expiratory ratio 1:2, and FiO₂ 40%–60%, adjusted to maintain end-tidal CO_2_ between 35 and 45 mmHg.

For hypotension management, MAP < 65 mmHg or a decrease of more than 20% from baseline prompted consideration of rescue therapy with fluid bolus (500 mL crystalloid) or vasopressors (phenylephrine 50–100 μg or ephedrine 5–10 mg), guided by the anaesthesiologist's clinical judgment.

Anesthesia was maintained with continuous intravenous infusion of propofol at 4–12 mg/kg/h and remifentanil at 0.1–0.3 μg/kg/min, keeping the BIS value between 40 and 60. During surgery, the infusion rates of propofol and remifentanil were adjusted according to the depth of anesthesia, and muscle relaxants were administered as needed.

### 2.4 Study outcomes

The primary outcomes were success rate of the first intubation attempt and intubation time (defined as the interval from insertion of the gastrointestinal endoscope through the bite block to connection of the breathing circuit to the endotracheal tube after successful intubation). Secondary outcomes included number of intubation attempts (withdrawal and readjustment counted as one attempt), hemodynamic stability (MAP, HR, SpO₂) at before induction of anesthesia (BA), post-induction (T1), post-intubation (T2), 3 min post-intubation (T3), and 10 min after tracheal intubation (T4). Peri-operative adverse events (esophageal intubation, lip/teeth injury, hoarseness, sore throat), Intubation failure (intubation time >2 min or more than 2 attempts). Patient characteristics and airway assessment parameters were recorded.

Postoperative sore throat and hoarseness were assessed by a research nurse who was blinded to the study objectives and the specific intubation technique, using a 4-point verbal rating scale (0 = none, 1 = mild, 2 = moderate, 3 = severe) (7). All patients remained hospitalised during the routine postoperative observation period following their endoscopic procedures, which typically exceeded 48 h. Any new-onset or persistent symptoms occurring during this period were documented by the ward nursing staff during daily clinical rounds and reported to the research team. Hemodynamic parameters were automatically recorded by the patient monitor, and adverse events were documented by the treating anesthesiologist at the time of the procedure. SpO₂ was recorded from the patient monitor at each of the five predefined timepoints (BA, T1, T2, T3, T4). These were spot readings at protocol-defined timepoints; continuous trend monitoring was not systematically captured for nadir determination. Full blinding of treating clinicians and participants was not applicable given the single-arm design.

Adverse events were ascertained through active surveillance, including direct observation by the treating anesthesiologist, real-time documentation in the anesthesia record, and systematic review of all case report forms (including a dedicated ‘other complications’ field) and anesthesia records at the end of the study period. The observation window extended from induction through the entire routine postoperative hospitalisation period (minimum 48 h for all patients), ensuring complete capture of pulmonary complications and other adverse events during this period.

The primary outcome definitions (first-attempt success rate and intubation time) were prespecified in the study protocol and recorded in the ClinicalTrials.gov registry (NCT06990490). The *a priori* failure definition (intubation time >2 min or >2 attempts; as defined in Section 2.3) was also prespecified in the study protocol prior to data collection; however, this specific threshold was not explicitly included in the registry record at the time of retrospective registration, nor was it added during the August 2025 registry update. We further confirm that this August 2025 update did not alter the primary outcome definitions.

Although the primary outcome (first-attempt success rate and intubation time) was prespecified in the clinical trial registry, the secondary outcomes—including hemodynamic parameters, adverse events, and procedural complications—were not explicitly prespecified and are therefore reported as *post-hoc* outcomes. The analysis plan for these outcomes was finalized prior to data analysis.

### 2.5 Statistical analysis

Normally distributed continuous variables are presented as mean ± standard deviation (SD), whereas non-normally distributed continuous variables are expressed as median (interquartile range). Categorical variables are summarized as frequencies (percentages). Repeated-measures analysis of variance (ANOVA) was used to compare hemodynamic parameters across five predefined time points (BA, T1, T2, T3, T4). The Greenhouse–Geisser correction was applied when the sphericity assumption was violated. For significant main effects, *post-hoc* tests with Bonferroni adjustment were performed to detect differences between time points. A *p*-value < 0.05 was defined as statistically significant.

Mauchly's test was performed to evaluate sphericity for each repeated-measures variable. Sphericity was violated for mean arterial pressure (MAP; Mauchly's W = 0.528, *p* = 0.005), so the Greenhouse–Geisser correction (*ε* = 0.762) was applied. For heart rate (HR), the sphericity assumption was satisfied (Mauchly's W = 0.836, *p* = 0.667), and uncorrected degrees of freedom were reported. Partial *η*^2^ was reported as the effect size for all ANOVA models. Confidence intervals for proportions were calculated using the exact (Clopper-Pearson) method. All statistical analyses were audited by an independent biostatistician.

## 3 Results

A total of 40 patients were enrolled in this study (Figure 2). Table 1 shows the patient demographic and clinical characteristics including age, gender, body mass index (BMI), American Society of Anesthesiologists (ASA) physical status, and airway assessment data.

**Figure 2:**
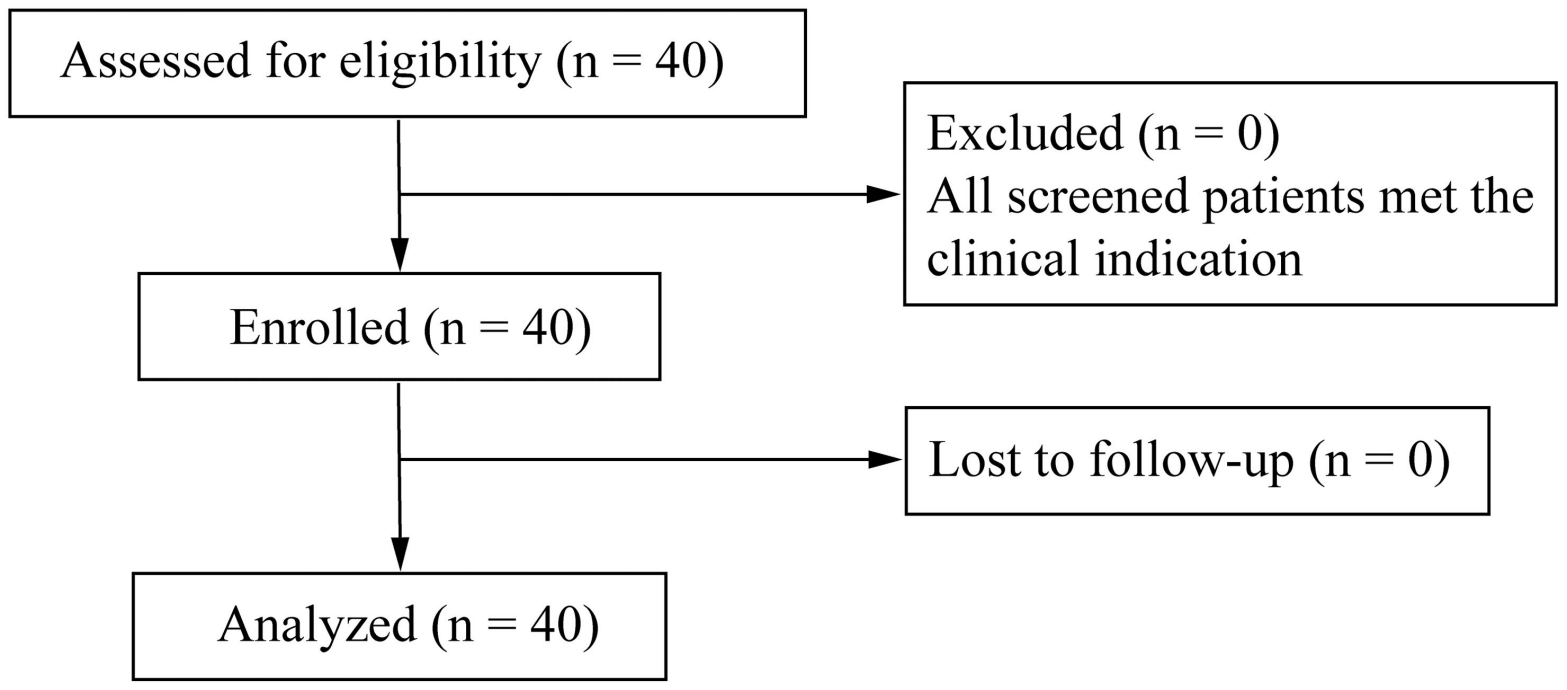
A total of 40 patients were screened, enrolled, and completed the study without any exclusions or loss to follow-up; all were analysed.

**Table 1:** Demographic and airway assessment data.

Characteristic	Patients (n=40)
Age, y	56.23 ± 15.52
Gender (M/F)	28 (70%)/12 (30%)
Body mass index, kg/m2	23.88 ± 4.3
ASA grade (Ⅰ/Ⅱ/Ⅲ) (n)	0/38 (95%)/2 (5%)
Mallampati score (1/2/3/4) (n)	11 (27.5%)/11 (27.5%)/15 (37.5%)/3 (7.5%)
Mouth opening, (≥3 cm/<3 cm)	40 (100%)/0
Thyromental distance, cm	7.79 ± 1.67
Jaw protrusion test (1/2/3) (n)	25 (62.5%)/11 (27.5%)/4 (10%)
Maxillofacial Morphology and Relevant Medical History	
Sleep apnea syndrome (SAS) (n)	4 (10%)
Ankylosing spondylitis (AS) (n)	1(2.5%)

Data are shown as *n* or mean ± SD or number of patients (%). Jaw protrusion was assessed using the jaw protrusion test (also known as the upper lip bite test): grade 1 = lower incisors can be protruded anterior to upper incisors (normal protrusion); grade 2 = lower incisors can be brought edge-to-edge but not anterior to upper incisors; grade 3 = lower incisors cannot be brought to the level of the upper incisors (severely limited protrusion).

The intubation time was 30.95 ± 6.58 s [median [IQR]: 31.00 [26.00–35.00] s; range: 17–45 s]. All 40 patients were successfully intubated on the first attempt, yielding a first-attempt success rate of 100% (95% CI: 91.2%–100%) (Table 2). No patient experienced esophageal intubation (0/40; 95% CI: 0%–8.8%), dental injury (0/40; 95% CI: 0%–8.8%), hypoxia (0/40; 95% CI: 0%–8.8%), or other severe complications (Table 3).

**Table 2:** Intubation parameters and success rates.

Parameter	Value
Intubation time, s	
Mean ± SD	30.95 ± 6.58
Median (IQR)	31.00 (26.00–35.00)
Range	17–45
Intubation, *n* (%)	
Success at 1st attempt	40 (100%)
Success at 2nd attempt	0
Esophageal intubation	0

Data are shown as mean ± SD, median (IQR), range, or number of patients (%). Confidence intervals for proportions are reported as two-sided 95% exact Clopper-Pearson intervals.

**Table 3:** Adverse effects and complications.

Complications	*n* (%)	95% CI
Oral mucosal injury	0	0%–8.8%
Dental injury	0	0%–8.8%
Sore throat	9 (22.5%)	11.0–38.2%
Hoarseness	0	0%–8.8%

Data are presented as number of patients (%). Confidence intervals for proportions are reported as two-sided 95% exact Clopper-Pearson intervals.

During the intubation period, no patient experienced desaturation (SpO₂ < 90%) at any of the five predefined timepoints. SpO₂ remained at 100% at all recorded timepoints (BA, T1, T2, T3, T4). All patients received 3 min of preoxygenation with 100% FiO₂ prior to induction, and the mean apnoeic interval was 30.95 ± 6.58 s (range: 17–45 s).

Mask ventilation in the lateral position was assessed clinically. All patients had mouth opening ≥ 3 cm (Table 1). Although 3 patients had a Mallampati class IV, no patient exhibited difficulty with mask ventilation during the induction period. No patient required a two-person technique for effective mask ventilation.

A jaw thrust manoeuvre was performed routinely in all 40 patients as part of the standard intubation technique. The endoscopic view of the glottis was clear and unobstructed in all cases, and no patient required repositioning of the endoscope or scope withdrawal during the procedure.

Following anesthesia induction, 15 out of 40 patients (37.5%) experienced a transient decrease in MAP below 65 mmHg; non-invasive blood pressure was measured at 2.5-minute intervals. By the post-intubation study time-point T2, MAP in all these patients had recovered to ≥65 mmHg. The exact duration of each hypotensive episode could not be determined given intermittent NIBP sampling. No vasopressors or rescue fluid boluses were administered for hypotension. This constitutes a protocol deviation: predefined MAP-based treatment thresholds were not strictly implemented, although this omission reflected clinical judgement, as hypotension was brief and unaccompanied by clinical markers of end-organ hypoperfusion.

In addition to the events listed in Table 3, systematic review of the ‘other complications’ field in all case report forms and anesthesia records confirmed no occurrences of regurgitation, aspiration, laryngospasm, bronchospasm, gastric insufflation, tube dislodgement or malposition during subsequent endoscopy, or postoperative pulmonary complications.

Hemodynamic data (MAP and HR) across the five timepoints are presented in Figure 3 and detailed numeric values are summarized in Table 4. Repeated-measures ANOVA revealed a statistically significant main effect of time on both MAP [F (3.048, 118.888) = 23.05, *p* < 0.001, partial *η*^2^ = 0.372] and HR [F (4, 156) = 3.258, *p* = 0.013, partial *η*^2^ = 0.077]. *Post-hoc* analysis confirmed the characteristic hemodynamic response to anesthesia induction and endotracheal intubation. For MAP, there was a significant decrease at T1 (post-induction) compared to baseline [mean difference: −14.70 mmHg, 95% CI (−20.79, −8.61), *p* < 0.001], followed by a significant increase at T2 (post-intubation) above T1 levels [mean difference: +18.58 mmHg, 95% CI (12.55, 24.61), *p* < 0.001]. For HR, repeated-measures ANOVA showed a significant main effect of time (*p* = 0.013, partial *η*^2^ = 0.077); however, pairwise comparisons revealed that HR changes from baseline did not reach statistical significance after Bonferroni adjustment at any time point (all *p* > 0.05). The significant time effect for HR was driven by a modest increase from T1 to T2 [mean difference: +6.13 bpm, 95% CI (0.37, 11.89), *p* = 0.030], reflecting a transient sympathetic response. Crucially, this hemodynamic stress response was transient. Both MAP and HR rapidly returned to levels statistically indistinguishable from baseline by the 3-minute post-intubation timepoint (T3) and remained so at T4 (all *p* > 0.05). The maximal increase from baseline occurred immediately post-intubation (T2), with an increase of +4.5% in MAP and +6.1% in HR. Complete Bonferroni-adjusted pairwise comparisons across all five timepoints for MAP and HR are presented in Table 5.

**Figure 3:**
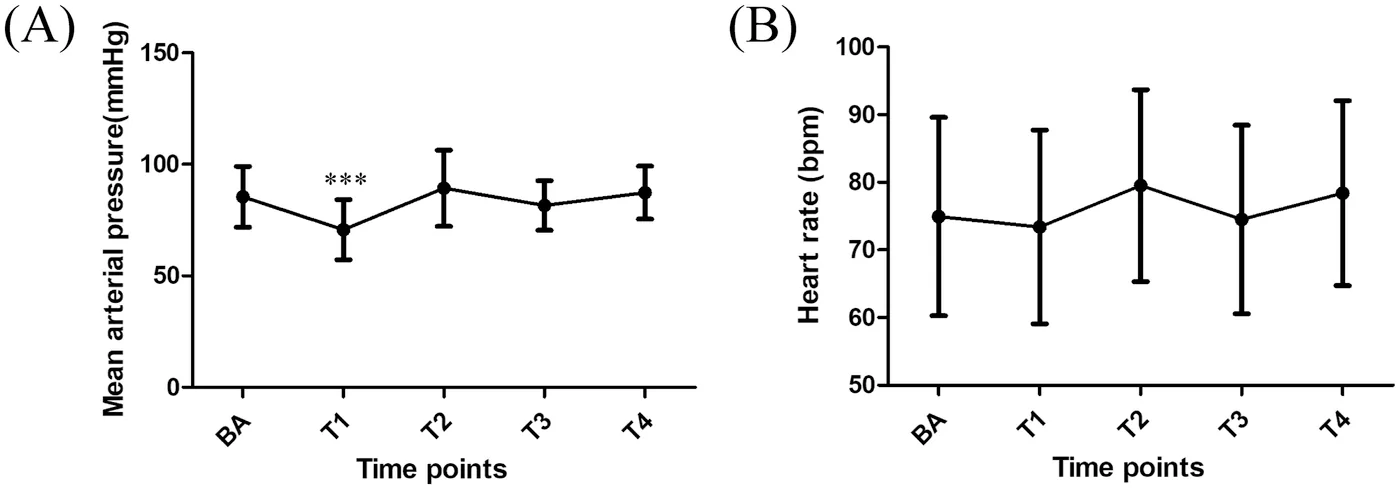
Serial perioperative hemodynamic fluctuations recorded at five predefined time points (BA, T1, T2, T3, T4). Data are presented as mean ± SD (*n* = 40). **(A)** Dynamic changes in mean arterial pressure (MAP, mmHg). **(B)** Dynamic changes in heart rate (HR, beats per minute, bpm). *p < 0.05, **p < 0.01, ***p < 0.001 vs. BA (Bonferroni-adjusted *post-hoc* comparisons). For HR, none of the pairwise comparisons versus baseline reached statistical significance after Bonferroni adjustment (all *p* > 0.05). The *y*-axis ranges were selected independently to optimally display the data for each variable. BA: before anesthesia induction; T1: post-induction; T2: immediate post-intubation; T3: 3 min post-intubation; T4: 10 min post-intubation.

**Table 4:** Perioperative hemodynamic and oxygenation parameters.

Time	MAP(mmHg)	HR(bpm)	SpO_2_(%)
BA	85.4 ± 13.61	74.95 ± 14.67	100
T1	70.7 ± 13.54	73.38 ± 14.33	100
Difference vs. BA	−14.70 [−20.79, −8.61], *p* < 0.001*	−1.57 [−8.67, 5.52], *p* = 1.000	-
T2	89.28 ± 17.05	79.5 ± 14.18	100
Difference vs. BA	+3.88 [−4.27, 12.02], *p* = 1.000	+4.55 [−2.13, 11.23], *p* = 0.494	-
T3	81.53 ± 11.11	74.5 ± 13.95	100
Difference vs. BA	−3.87 [−10.36, 2.61], *p* = 0.832	−0.45 [−6.99, 6.09], *p* = 1.000	-
T4	87.3 ± 11.86	78.4 ± 13.66	100
Difference vs. BA	+1.90 [−4.42, 8.22], *p* = 1.000	+3.45 [−2.59, 9.49], *p* = 0.969	-

Data are mean ± SD or mean difference [95% CI], Bonferroni-adjusted. BA: before anesthesia induction; T1: post-induction; T2: immediate post-intubation; T3: 3 min post-intubation; T4: 10 min post-intubation. Repeated-measures ANOVA: MAP: F(3.048, 118.888) = 23.05, *p* < 0.001, partial *η*^2^ = 0.372; HR: F(4, 156) = 3.258, *p* = 0.013, partial *η*^2^ = 0.077. SpO₂ was 100% at each of the five predefined timepoints in all patients; continuous trend data were not captured. *p < 0.05 vs. BA.

**Table 5:** Bonferroni-adjusted pairwise comparisons for MAP and HR.

Comparison	MAP Mean difference (mmHg) [95% CI]	Adjusted *p*	HR Mean difference (bpm) [95% CI]	Adjusted *p*
T1 vs. BA	−14.70 [−20.79, −8.61]	<0.001	−1.57 [−8.67, 5.52]	1.000
T2 vs. BA	+3.88 [−4.27, 12.02]	1.000	+4.55 [−2.13, 11.23]	0.494
T3 vs. BA	−3.87 [−10.36, 2.61]	0.832	−0.45 [−6.99, 6.09]	1.000
T4 vs. BA	+1.90 [−4.42, 8.22]	1.000	+3.45 [−2.59, 9.49]	0.969
T2 vs. T1	+18.58 [12.55, 24.61]	<0.001	+6.13 [0.37, 11.89]	0.030
T3 vs. T1	+10.83 [5.98, 15.67]	<0.001	+1.13 [−4.53, 6.78]	1.000
T4 vs. T1	+16.60 [10.19, 23.01]	<0.001	+5.03 [−1.23, 11.28]	0.218
T3 vs. T2	−7.75 [−13.69, −1.81]	0.004	−5.00 [−10.28, 0.28]	0.075
T4 vs. T2	−1.98 [−10.23, 6.28]	1.000	−1.10 [−7.65, 5.45]	1.000
T4 vs. T3	+5.78 [0.58, 10.97]	0.020	+3.90 [−1.89, 9.69]	0.520

Data are mean differences [95% CI], Bonferroni-adjusted. BA: before anesthesia induction; T1: post-induction; T2: immediate post-intubation; T3: 3 min post-intubation; T4: 10 min post-intubation.

## 4 Discussion

Endotracheal intubation serves as the core airway management technique in clinical anesthesia, with the supine position being the standard posture for routine intubation owing to its optimal laryngopharyngeal anatomical alignment and familiar operating posture for anesthesiologists. Nevertheless, a large number of gastrointestinal endoscopic diagnostic and therapeutic procedures require patients to maintain the left lateral decubitus position throughout anesthesia. During general anesthesia with tracheal intubation for endoscopy, the endoscopist operates at the patient's head side; frequent adjustment and movement of the gastrointestinal endoscope may loosen the fixation device of the orotracheal tube, which carries a potential risk of unexpected tube dislodgement and acute airway loss. In this context, establishing a safe, efficient and stable tracheal intubation technique suitable for patients in the lateral decubitus position is of great clinical significance for perioperative anesthesiologists.

The present prospective feasibility cohort study enrolled 40 patients undergoing elective gastrointestinal endoscopy in the left lateral decubitus position, and systematically evaluated the feasibility, safety and hemodynamic characteristics of gastrointestinal endoscopy-guided endotracheal intubation. Our results demonstrated a 100% first-attempt intubation success rate with a mean intubation time of 30.95 ± 6.58 s, and no severe perioperative airway-related complications were observed, which supported the feasibility of this approach as a technique that merits further investigation for patients receiving lateral-positioned endoscopic interventions.

The lateral decubitus position brings unique challenges to tracheal intubation, mainly including distorted airway anatomical structure and unfamiliar operating posture for clinicians. Even so, lateral-position intubation possesses irreplaceable perioperative application value. It can be applied to artificial airway establishment for patients with mandatory lateral positioning, emergency airway rescue for intraoperative respiratory depression, and management of accidental tracheal tube slippage during surgery (8, 9). For patients with a full stomach, the lateral position itself facilitates the clearance of oropharyngeal secretions by gravity, and lateral mask ventilation produces lower peak inspiratory pressure than supine ventilation, together reducing the risk of aspiration (10, 11). Our technique adds a protective mechanism: the endoscope provides continuous real-time visualization of the pharynx and glottic area, allowing immediate detection and suctioning of regurgitation while maintaining glottic observation—a capability not available with conventional laryngoscopy. In this cohort, no aspiration or regurgitation events were documented. However, we acknowledge that the absence of such events in 40 patients does not permit definitive conclusions about the true incidence of these general anaesthesia-related complications.

### 4.1 Key findings and hemodynamic profile

A notable finding of this technique was its procedural efficiency coupled with mild, transient hemodynamic disturbance. Repeated-measures ANOVA showed that time exerted a significant main effect on both MAP [F (3.048, 118.888) = 23.05, *p* < 0.001, partial *η*^2^ = 0.372] and HR [F (4, 156) = 3.258, *p* = 0.013, partial *η*^2^ = 0.077]. Consistent with previous research, tracheal intubation acts as a powerful nociceptive stimulus that activates the sympathetic nervous system, triggering transient tachycardia and hypertension (12–14). In our cohort, MAP and HR increased by 4.5% and 6.1% relative to baseline immediately after intubation (T2), respectively. For MAP, the change from baseline was statistically significant at T1 (post-induction, *p* < 0.001) and returned to baseline levels by T3 (all *p* > 0.05 for T3 and T4 vs. BA). For HR, although the repeated-measures ANOVA showed a significant time effect (*p* = 0.013, partial *η*^2^ = 0.077), pairwise comparisons against baseline did not reach statistical significance at any time point after Bonferroni adjustment. The significant HR response was detected as an increase from T1 to T2 [mean difference: +6.13 bpm, 95% CI (0.37, 11.89), *p* = 0.030], indicating a transient sympathetic response that was modest in clinical magnitude. Both MAP and HR recovered to baseline levels by the 3-minute post-intubation timepoint (T3) and remained stable at T4. Although the repeated-measures ANOVA indicated a statistically significant effect of time on both MAP and HR, the maximal observed increases from baseline were modest in clinical terms (MAP +4.5%, HR +6.1%). Both parameters returned to baseline levels within 3 min, suggesting that the haemodynamic response to this intubation technique is not only transient but also clinically well-tolerated.

The induction regimen may have attenuated the haemodynamic response to intubation, representing a potential confounder. Midazolam may also affect BIS interpretation, as benzodiazepines influence the bispectral index independently of hypnotic depth. However, BIS was used as an ancillary monitor alongside clinical parameters.

Although direct comparisons are not possible due to differences in study populations, anesthetic regimens, and outcome definitions, the hemodynamic changes observed in our cohort appeared to recover more rapidly than those reported in previous FOB-guided intubation studies (15) and appeared milder than those described for conventional direct laryngoscopy (12, 13). However, these cross-study comparisons are hypothesis-generating only and require confirmation in future head-to-head trials. It should also be noted that the intubation time definition used in this study—from endoscope insertion to circuit connection—differs from those in some comparator studies, where intubation time was measured from laryngoscope/blade insertion to capnographic confirmation (10, 15). This difference should be considered when interpreting cross-study comparisons. We acknowledge that we did not separately record the interval from device insertion to the first capnograph waveform, as this was not a prespecified outcome. The intubation time reported here represents the total apnoeic interval from endoscope insertion to circuit connection, which was our predefined primary metric.

### 4.2 Comparison with existing airway management techniques

The core innovation of our protocol lies in repurposing standard gastrointestinal endoscopes—universally available equipment in endoscopy suites—as the primary visualization tool for tracheal intubation, which fundamentally distinguishes it from traditional FOB-guided intubation in two critical dimensions. First, this method may reduce reliance on specialized airway equipment such as fiberoptic bronchoscopes, which are not readily accessible in many gastroenterology operating rooms, potentially simplifying on-site logistics and reducing equipment costs. Second, we preload a stiff stylet into the reinforced endotracheal tube; the endoscopist advances the gastrointestinal endoscope through the central channel of the bite block, while the anesthesiologist delivers the tracheal tube via the bite block's right lateral port. The stiff stylet provides robust rigidity and directional control, facilitating tube advancement along the visualized trajectory toward the glottis, which may help minimize the risk of esophageal misintubation.

When contrasted with video laryngoscopy performed in the lateral position, our technique enables continuous, full-length real-time visualization of the entire airway tract from the oral cavity to the trachea, as the endoscope provides a dynamic view that follows the anatomical contours. This may offer advantages over video laryngoscopy, which provides a fixed-angle view that can be limited by anatomical constraints in the lateral position.

The most directly comparable prior study is a randomized controlled trial by Jin et al., which compared video laryngoscopy-guided intubation in the left lateral position versus the supine position during upper gastrointestinal endoscopic surgery in 120 patients (10). Their findings demonstrated that intubation in the lateral position under video laryngoscopy was feasible and safe, with comparable intubation time (23.95 ± 4.43 s vs. 23.44 ± 4.78 s, *P* = 0.545) and 100% success rates in both groups, while significantly reducing hemodynamic fluctuations and the severity of sore throat. Additionally, they observed that mask ventilation in the lateral position was associated with significantly lower peak inspiratory pressure than in the supine position (discussed further in Section 4.3). Our technique differs from video laryngoscopy in that it provides continuous, real-time endoscopic visualization throughout the entire intubation process, rather than relying on a fixed blade angle that may be susceptible to line-of-sight obstruction. However, we acknowledge that formal head-to-head comparative studies are needed to determine whether this technique offers advantages over video laryngoscopy. These cross-study comparisons are hypothesis-generating only and should not be interpreted as evidence of superiority.

We acknowledge the contribution of Barakat et al., who conducted a prospective randomized trial comparing endoscopist-facilitated intubation—using an endotracheal tube backloaded onto an ultra-slim gastroscope—with standard intubation for ERCP (5). Their study demonstrated a median intubation time of 0.63 min and a significantly lower incidence of post-procedure throat discomfort (13% vs. 50%, *p* < 0.01) in the endoscopist-facilitated group. However, their approach differs from ours in a key technical aspect: the tube is backloaded onto the endoscope itself (similar to a fiberoptic bronchoscope-guided technique), whereas our method employs simultaneous but independent insertion of the endoscope and tube through separate channels of a bite block. This distinction may offer greater flexibility in tube manipulation and may allow the endoscope to remain immediately available for the subsequent endoscopic procedure without the need for reloading or reinsertion.

The clinical utility of this technique is further supported by a recent case report from our institution, which successfully employed a closely related approach for airway management in a patient with massive variceal bleeding and hypovolemic shock (6). In that emergency scenario, the technique enabled rapid first-pass intubation under direct endoscopic visualization, avoided repositioning-induced hemodynamic instability, allowed seamless transition to immediate endoscopic hemostasis, and was not associated with visible aspiration or mucosal injury. Of note, the case report and the present study are independent investigations with no patient or text overlap; the case report involved a single emergency patient, whereas our study provides prospective data from a consecutive cohort of 40 elective patients. Collectively, these observations suggest the technique may be applicable across a spectrum of clinical settings, from elective procedures to emergencies. However, formal head-to-head comparative studies are needed to establish its relative advantages.

### 4.3 Multidisciplinary synergy and anatomical advantages of the lateral position

From an anatomical perspective, the left lateral decubitus position presents a duality of effects on airway anatomy. On one hand, it distorts the normal oro-pharyngeal-laryngeal axis compared with the supine position, which can increase the technical difficulty of direct laryngoscopy (2, 3). On the other hand, gravity pulls the tongue and epiglottis laterally, mitigating tongue base collapse against the posterior pharyngeal wall, expanding the pharyngeal cavity, and facilitating mask ventilation. Indeed, Jin et al. observed that mask ventilation in the lateral position was associated with lower peak inspiratory pressure than in the supine position (10). This dual effect—greater distortion for direct laryngoscopy, yet potential advantages for endoscopic approaches—explains why intubation in the lateral position remains challenging with conventional techniques but may be feasible with visualization tools that can adapt to the altered anatomy. In our approach, the gastrointestinal endoscope provides a dynamic, real-time view that follows the anatomical contours of the oropharynx and larynx, making it particularly suited to the lateral position where fixed-angle devices may struggle.

Furthermore, close multidisciplinary collaboration between anesthesiologists and gastroenterologists may generate synergistic effects. The gastrointestinal endoscope fulfills dual functions: it provides clear visualization to facilitate tracheal intubation, and can be immediately shifted to perform diagnostic or therapeutic endoscopic procedures without instrument replacement or patient repositioning.

This integrated workflow may streamline the overall procedure, may reduce the risk of accidental extubation or tube displacement during positional transfers, and may reduce the need for additional personnel to reposition the patient. However, these potential benefits were not formally quantified in the present study and require comparative evaluation in future time-and-motion or total procedure-time analyses. It should be noted, however, that the technique requires the simultaneous presence of both an anesthesiologist and a gastroenterologist during the intubation procedure—a different staffing model than standard single-operator laryngoscopic intubation. In the endoscopy suite setting, the gastroenterologist is already present for the subsequent procedure and simply initiates their role earlier; the same endoscope is then advanced immediately after tube placement to perform the endoscopic examination. The net staffing impact should be formally evaluated in future time-and-motion studies.

This integrated workflow may be particularly valuable in high-risk patients. We did not exclude patients based on ASA status because this technique is the standard of care in our department for lateral-position procedures. In patients with higher ASA grades or hemodynamic instability, lateral-position intubation may offer a distinct advantage—by avoiding post-induction repositioning and the associated hemodynamic fluctuations—over conventional supine intubation.

An important practical consideration is infection control. In our workflow, the same gastrointestinal endoscope is used first to guide tracheal intubation (with the scope positioned at the glottic level) and then advanced into the oesophagus and stomach for the endoscopic procedure. No intervening cleaning or disinfection step is performed between these two phases, as the scope remains within the patient's upper aerodigestive tract and does not leave the patient. Importantly, the theoretical risk of translocating oropharyngeal flora into the upper gastrointestinal tract is no different from that associated with routine diagnostic or therapeutic endoscopy, as oropharyngeal suctioning is performed during both procedures to maintain a clear visual field. All endoscopes undergo standard high-level disinfection after each procedure according to institutional protocols, which is identical to the reprocessing performed for routine endoscopic examinations. In this cohort, no infectious complications were observed.

Another important practical consideration is the potential for endoscope damage during the intubation procedure. Based on unaudited institutional experience over many years of clinical use, we have not observed any instance of endoscope damage attributable to this technique; however, this observation is derived from informal clinical experience rather than systematically collected safety data. The stylet is preloaded into the endotracheal tube without exceeding the distal port, and the tube is advanced through the separate right lateral channel of the bite block while the endoscope passes through the central channel, maintaining physical separation. Continuous endoscopic visualization further minimizes the risk of inadvertent contact. Regarding cost implications, the technique utilizes the same endoscope already required for the subsequent endoscopic procedure. This may reduce reliance on dedicated advanced airway equipment for the initial intubation in appropriately equipped endoscopy suites; however, conventional and rescue airway equipment remains necessary, as reflected by the study's own rescue protocol. The integrated workflow may reduce overall procedure time compared to sequential approaches, and no additional reprocessing cycles are introduced. Formal cost-effectiveness analysis would be valuable to quantify these trade-offs in future studies.

In our cohort, the incidence of mild postoperative sore throat was 22.5%, and all symptoms resolved spontaneously within 24 h. No severe complications including dental trauma, mucosal bleeding, laryngospasm, laryngeal edema, esophageal intubation or hypoxia occurred in this study.

### 4.4 Clinical experience with difficult airways

Although the formal prospective data of this research only included 40 enrolled patients, our department has accumulated considerable clinical experience with this technique over many years, although this experience has not been formally audited. In the current cohort, all patients with classic difficult intubation predictors (Mallampati class IV, obstructive sleep apnea, ankylosing spondylitis, etc.) achieved successful first-attempt intubation. This technique requires transoral gastroscope passage; therefore, patients with severely restricted mouth opening that precluded safe endoscope insertion were not candidates for this approach. Consistent with this, all enrolled patients in our cohort had mouth opening ≥ 3 cm. The magnified, continuous endoscopic view of the glottis may help overcome certain anatomical obstacles associated with conventional laryngoscopy, such as poor cervical spine mobility and bulky tongue base. However, we acknowledge that all patients in this cohort had mouth opening ≥ 3 cm, and therefore our data do not support a conclusion that the technique is effective in patients with restricted mouth opening. The two-channel bite block may actually require greater mouth opening than direct laryngoscopy, and this should be evaluated in future studies that specifically enroll patients with limited mouth opening.

### 4.5 Study limitations

Several inherent limitations of this trial should be objectively acknowledged. First, despite the support of long-term institutional clinical experience, the quantitative data analyzed in this study were derived from a small sample of only 40 patients, lacking sufficient statistical power for subgroup analysis. Second, all intubation procedures were completed by one fixed skilled anesthesiologist and one specialized gastroenterologist at a single tertiary hospital. The operation proficiency and mature multidisciplinary cooperation mode in our center cannot be fully replicated in primary medical institutions or teams with less experienced operators, which restricts the generalizability of our findings. Third, although several difficult airway patients were included, this trial was not specially designed or powered to evaluate the efficacy of this technique in a dedicated difficult-airway cohort; thus, we cannot draw definitive conclusions that this method outperforms traditional airway tools in high-risk difficult airway populations. Fourth, although the observed first-attempt success rate was 100% in our cohort of 40 patients, the exact binomial 95% confidence interval (91.2%–100%) indicates that the true success rate in the broader population could be as low as approximately 91%. This statistical uncertainty should be considered when interpreting the strength of our findings, and larger studies are needed to provide more precise estimates of the technique's success rate. Fifth, all procedures were performed by one fixed anesthesiologist-gastroenterologist pair. The learning curve for this technique has not been formally evaluated. Although our institutional experience suggests that the technique is relatively easy to learn, these observations are anecdotal and have not been systematically studied. Future studies should formally evaluate the learning curve and assess the performance of this technique across operators with varying levels of experience. Sixth, the ASA distribution in our cohort was predominantly ASA II (38/40), with only 2 ASA III patients and no ASA IV + patients. While we did not exclude patients based on ASA status, the actual enrolled population reflects a relatively low-risk cohort, which limits the generalizability of our findings to higher-risk populations with more severe comorbidities. Future studies should evaluate the performance of this technique in ASA IV + patients. Seventh, the study was registered retrospectively. Data collection was completed on March 31, 2025, and registration was completed on May 10, 2025—after data collection had finished. The registry entry was subsequently updated in August 2025 to correct administrative errors in the study timeline dates and to add secondary outcomes that had been prespecified in the analysis plan prior to data analysis; the primary outcome definitions and the *a priori* failure definition were not altered in this update. We have mitigated the potential for selective outcome reporting by prespecifying the analysis plan for all outcomes prior to data analysis, as noted in the Methods section. Eighth, a transient hypotensive response following induction (MAP < 65 mmHg) was observed in 15 out of 40 patients (37.5%). This finding is most plausibly attributable to the induction regimen (midazolam, etomidate, sufentanil, and atracurium) rather than to the intubation technique itself. MAP recovered to ≥65 mmHg by the post-intubation study time-point T2, and no vasopressor agents were administered; exact episode duration could not be quantified due to intermittent NIBP sampling. These 15 patients met predefined study-protocol hypotension treatment thresholds but did not receive rescue therapy, constituting a protocol deviation guided by real-time clinical judgement. Ninth, SpO₂ was recorded as spot readings at five predefined timepoints rather than through continuous pulse-oximetry trend capture with systematic nadir extraction, as described in the Methods section. While no desaturation below 90% was observed at any recorded timepoint, we cannot provide the absolute lowest SpO₂ value throughout the entire intubation period. Tenth, tracheal tube cuff pressure was maintained within the recommended range (20–30 cmH₂O) using a handheld manometer, and tube size was selected based on patient sex according to standard clinical practice, as described in the Methods section; however, individual cuff pressure values and specific tube sizes per patient were not systematically recorded, as these were not prespecified outcomes of this feasibility study.

### 4.6 Clinical recommendations and future directions

Based on these preliminary feasibility data, this technique appears to be a safe and efficient option for airway management in patients undergoing left lateral decubitus gastrointestinal endoscopy. However, given the uncontrolled, single-center design with a limited sample size and expert operators, we emphasize that these findings are preliminary. The technique should be considered a promising approach that warrants further evaluation in larger, multicenter prospective trials with head-to-head comparisons against established airway devices before routine adoption can be recommended.

To further validate the reproducibility and wide applicability of this technique, large-sample, multicenter prospective trials are urgently required. Subsequent research should recruit independent cohorts of patients with confirmed difficult airways, and set up head-to-head control groups with video laryngoscopy and flexible fiberoptic bronchoscopy to comprehensively compare primary endpoints (intubation success rate, operation time) and secondary endpoints (hemodynamic stability, postoperative complication spectrum) across diverse clinical settings.

## 5 Conclusion

In this prospective feasibility cohort of 40 patients undergoing elective gastrointestinal endoscopic surgery in the left lateral decubitus position, gastrointestinal endoscopy-guided endotracheal intubation demonstrated a 100% first-attempt success rate, a mean intubation time of 30.95 ± 6.58 s, and a favorable hemodynamic profile (noting that 37.5% of patients experienced transient post-induction hypotension; MAP had returned to ≥65 mmHg by the post-intubation measurement and no vasopressor agents were administered; this finding is most plausibly attributable to the induction regimen rather than to the intubation technique itself). No severe complications occurred in this cohort; however, the study is not powered to characterise complications occurring at rates below approximately 8.8%, and definitive safety conclusions cannot be drawn from this single-center feasibility cohort. These preliminary findings suggest that the technique is a promising approach that merits further investigation in larger, multicenter prospective studies with head-to-head comparisons against established airway devices.

## Data Availability

The original contributions presented in the study are included in the article/Supplementary Material, further inquiries can be directed to the corresponding author.

## Supplementary material

The Supplementary Material for this article can be found online at: https://www.frontiersin.org/articles/10.3389/fmed.2026.1934908/full#supplementary-material

Supplementary Video 1 Endoscopic-view footage of endotracheal intubation guided by gastrointestinal endoscope in a patient in the left lateral decubitus position.

Supplementary Video 2 Additional endoscopic-view footage of the same technique in another patient in the left lateral decubitus position.

Supplementary File 1 STROBE Statement checklist.

Supplementary File 2 Raw study data files.
