# Supplementary material for: A novel endoscopy-guided airway management strategy for lateral decubitus endoscopic procedures
Source: Front Med (Lausanne). 2026 Oct 13;13:1934908. doi: 10.3389/fmed.2026.1934908 (PMC13710366; doi:10.3389/fmed.2026.1934908)
Supplement: Supplementary file 1 [file Datasheet1.pdf]

ID	Age (years)	Sex	BMI	ASA grade	Positioning time (s)	Oropharyngeal airway used	Difficult mask ventilation	Number of attempts	Intubation time (s)
1	15	Male	19.1	II	75	No	No	1	35
2	40	Male	22.9	III	43	No	No	1	32
3	73	Male	24.9	II	65	No	No	1	37
4	46	Female	21.2	II	38	No	No	1	30
5	37	Female	18	II	41	No	No	1	30
6	56	Male	27.1	II	81	No	No	1	45
7	64	Female	23.2	II	35	No	No	1	31
8	58	Male	25.4	II	70	No	No	1	34
9	58	Male	24.2	II	25	No	No	1	17
10	63	Male	23.8	II	25	No	No	1	28
11	17	Female	17.3	II	85	No	No	1	31
12	36	Female	19.8	II	43	No	No	1	36
13	52	Male	24	II	25	No	No	1	33
14	77	Male	26.1	II	48	No	No	1	16
15	67	Female	23.9	II	58	No	No	1	28
16	71	Male	28.7	II	65	Yes	No	1	32
17	69	Male	22.3	II	70	No	No	1	22
18	67	Female	24	II	45	No	No	1	26
19	56	Male	24.2	II	35	No	No	1	26
20	59	Male	25.1	II	35	No	No	1	26
21	38	Male	28.4	II	33	No	No	1	40
22	62	Female	19.2	II	22	No	No	1	36
23	62	Male	20.9	II	93	No	No	1	22
24	61	Female	19.6	II	43	No	No	1	28
25	52	Female	17.3	II	33	No	No	1	31
26	83	Male	19.5	II	69	No	No	1	22
27	46	Male	24.6	II	43	No	No	1	35
28	36	Male	29.4	II	25	No	No	1	23
29	40	Male	21.2	II	35	No	No	1	25
30	63	Male	29.1	II	38	No	No	1	25
31	41	Female	33.2	II	35	No	No	1	43
32	66	Male	28.4	III	70	No	No	1	40
33	52	Male	37.6	II	65	No	No	1	34
34	64	Male	27.1	II	50	No	No	1	31
35	77	Male	19.1	II	53	No	No	1	35
36	78	Male	22.1	II	53	No	No	1	41
37	68	Male	26.1	II	65	No	No	1	35
38	67	Female	23.4	II	55	No	No	1	33
39	56	Male	22	II	58	No	No	1	31
40	56	Male	21.6	II	55	No	No	1	33

Baseline HR (bpm)	Baseline MAP (mmHg)	HR after induction (bpm)	MAP after induction (mmHg)	HR post-intubation (bpm)	MAP post-intubation (mmHg)
98	72	97	53	104	58
77	88	68	65	69	82
63	76	69	67	82	90
69	80	109	64	76	78
81	76	63	53	71	67
61	86	54	69	51	73
55	96	59	82	60	94
65	114	69	80	75	82
48	56	74	55	74	72
63	91	60	101	70	109
109	75	58	46	95	94
92	101	92	83	97	90
95	83	92	96	95	138
87	94	51	56	57	77
88	96	80	69	81	71
61	79	65	79	73	73
81	82	74	64	70	89
87	80	70	56	85	77
63	96	70	71	74	78
85	98	75	64	64	94
94	99	82	66	83	79
60	73	74	55	90	92
71	98	64	95	87	107
65	79	70	56	69	95
84	97	85	64	95	98
69	63	66	57	75	82
73	79	79	74	75	97
100	99	110	93	115	119
93	71	82	76	81	87
61	85	54	71	67	87
55	79	62	78	86	108
82	120	89	94	90	130
90	90	79	84	89	95
70	73	76	76	93	104
68	84	80	59	59	78
58	74	45	71	51	64
65	71	60	65	90	96
67	66	76	62	82	70
80	102	73	82	90	104
65	95	80	77	90	93

HR at 3 min post-intubation (bpm)	MAP at 3 min post-intubation (mmHg)	HR at 10 min post-intubation (bpm)	MAP at 10 min post-intubation (mmHg)	Medical history	Upper lip bite test
93	63	98	68	None	Class 2
53	79	80	94	Sleep apnea	Class 1
78	92	66	92	None	Class 2
80	82	102	99	None	Class 2
81	63	68	62	None	Class 1
53	74	79	88	None	Class 3
56	102	55	91	None	Class 2
75	85	83	109	None	Class 1
58	89	54	88	None	Class 1
76	104	73	114	None	Class 1
75	76	85	72	None	Class 1
96	80	89	96	None	Class 1
93	105	85	93	None	Class 3
51	71	79	89	None	Class 1
85	87	94	97	None	Class 2
54	71	64	82	Sleep apnea	Class 3
89	88	109	94	None	Class 1
75	78	90	92	None	Class 1
75	89	68	69	None	Class 1
82	82	59	85	None	Class 1
69	64	90	95	None	Class 1
75	82	84	78	None	Class 1
69	95	63	95	None	Class 1
62	73	62	89	None	Class 1
77	72	95	75	None	Class 2
73	71	83	87	None	Class 2
83	82	76	78	None	Class 1
120	95	109	94	None	Class 1
81	72	79	56	Ankylosing spondylitis	Class 2
65	80	81	103	Sleep apnea	Class 3
88	85	75	88	None	Class 1
71	106	73	98	None	Class 1
88	79	85	83	None	Class 2
81	82	80	90	None	Class 1
57	83	65	96	None	Class 1
65	69	59	78	None	Class 1
66	69	75	78	Sleep apnea	Class 2
75	76	78	81	None	Class 1
70	81	75	93	None	Class 1
67	85	69	83	None	Class 2

Mallampati class	Thyromental distance (cm)	Mouth opening 1≧3cm; 2＜3cm	Neck mobility 1>90°; 2<80°	Mandible length (cm)	Neck circumference (cm)
Class I	10	1	1	13	32
Class 3	9	1	1	16	35
Class 2	8	1	2	15	40
Class 3	8	1	1	13	30
Class I	9	1	1	12	26
Class 3	7	1	1	13	38
Class 3	7	1	1	15	32
Class 2	7	1	1	15	39
Class I	8	1	1	15	39
Class I	8	1	1	17	36
Class I	6	1	1	14	33
Class 3	7.5	1	1	13	30
Class 4	7.5	1	1	16	39
Class I	8	1	1	17	42
Class 3	6	1	1	15	41
Class 4	9	1	1	17	46
Class 3	6.5	1	1	13	33
Class 3	4	1	1	14	33
Class 2	8	1	1	17	38
Class 2	9.5	1	1	18	39
Class 2	8	1	1	16	38
Class 3	9	1	1	13	28
Class I	7	1	1	14	35
Class I	6	1	1	13	29
Class 3	6	1	1	15	30
Class 3	8	1	1	16	36
Class 2	8	1	1	16	37
Class 4	5.5	1	1	15	44
Class 3	7	1	2	15	33
Class 3	7	1	1	16	41
Class 2	10	1	1	12	38
Class 3	4	1	1	8	40
Class 2	9	1	1	13	33
Class 2	12	1	1	13	38
Class 2	8	1	1	11	33
Class 2	11	1	1	13	32
Class 3	9	1	1	15	39
Class I	10	1	1	13	28
Class I	7	1	1	10	36
Class I	7	1	1	10	38

Esophageal intubation	Lip/dental injury	Hoarseness	Sore throat
No	No	No	No
No	No	No	No
No	No	No	No
No	No	No	No
No	No	No	No
No	No	No	No
No	No	No	No
No	No	No	No
No	No	No	No
No	No	No	Yes
No	No	No	No
No	No	No	Yes
No	No	No	No
No	No	No	No
No	No	No	Yes
No	No	No	No
No	No	No	No
No	No	No	Yes
No	No	No	No
No	No	No	Yes
No	No	No	Yes
No	No	No	No
No	No	No	No
No	No	No	No
No	No	No	No
No	No	No	No
No	No	No	No
No	No	No	No
No	No	No	No
No	No	No	No
No	No	No	Yes
No	No	No	No
No	No	No	No
No	No	No	Yes
No	No	No	No
No	No	No	No
No	No	No	No
No	No	No	No
No	No	No	No
No	No	No	Yes
