# Supplementary material for: A novel endoscopy-guided airway management strategy for lateral decubitus endoscopic procedures
Source: Front Med (Lausanne). 2026 Oct 13;13:1934908. doi: 10.3389/fmed.2026.1934908 (PMC13710366; doi:10.3389/fmed.2026.1934908)
Supplement: Supplementary file 2 [file Table1.doc]

STROBE Statement—Checklist of items that should be included in reports of ***cohort studies***

|  | Item No | Recommendation |
| --- | --- | --- |
| **Title and abstract** | 1 | (*a*) Yes. Title: "A Novel Endoscopy-Guided Airway Management Strategy..." (feasibility implied). Abstract: "feasibility cohort" (Conclusion section) |
|  |  | (*b*) Yes. Abstract: Background, Methods, Results, and Conclusion sections. |
| Introduction | | |
| Background/rationale | 2 | Yes. Introduction: Paragraphs 1–2. Rationale for lateral-position intubation and endoscopic visualization described. |
| Objectives | 3 | Yes. Introduction: Last paragraph. "It was hypothesized that this novel technique is feasible, safe, and advantageous..." |
| Methods | | |
| Study design | 4 | Yes. Methods, section 2.1. "This was a prospective feasibility cohort study..." |
| Setting | 5 | Yes. Methods, section 2.1. Setting, hospital, dates (February–March 2025), data collection completion, and submission timeline described. |
| Participants | 6 | (a) Yes. Methods, section 2.2. Consecutive enrolment described; inclusion/exclusion criteria specified. Methods, section 2.4. 24-hour postoperative follow-up described. |
|  |  | (b) NA. Single-arm cohort study; no matching was performed. |
| Variables | 7 | Yes. Methods, section 2.4. Primary and secondary outcomes defined. Confounders (anaesthetic regimen) discussed in Discussion 4.1. |
| Data sources/ measurement | 8* | Yes. Methods, sections 2.3–2.4. Intubation time defined; haemodynamic measurements specified; adverse events/sore throat assessment methods described (blinded research nurse, 4-point scale). |
| Bias | 9 | Yes. Methods, section 2.4. Blinded research nurse for sore throat/hoarseness assessment; haemodynamic parameters automatically recorded; adverse events documented by treating anaesthesiologist. Full blinding not applicable given single-arm design. |
| Study size | 10 | Yes. Methods, section 2.2. "As this investigation was designed as a feasibility study, no formal a priori sample size calculation was performed. The sample size of 40 was chosen to provide a preliminary evaluation..." |
| Quantitative variables | 11 | Yes. Methods, section 2.5. "Normally distributed continuous variables are presented as mean ± SD, whereas non-normally distributed continuous variables are expressed as median (IQR)." |
| Statistical methods | 12 | (a) Yes. Methods, section 2.5. Repeated-measures ANOVA, Greenhouse-Geisser correction, Bonferroni post-hoc tests, effect sizes (partial η²), Clopper-Pearson CIs. |
|  |  | (b) NA. No subgroup analyses were performed due to limited sample size. |
|  |  | (c) Yes. No missing data. All 40 patients completed the study with complete data (Results, Figure 2). |
|  |  | (d) Yes. Results, Figure 2. No loss to follow-up (n=40 analysed). |
|  |  | (e) NA. No sensitivity analyses performed. |
| Results | | |
| Participants | 13* | (a) Yes. Results, Figure 2. 40 screened, 40 enrolled, 40 analysed. |
|  |  | (b) Yes. Methods, section 2.2. None were excluded; all screened patients met clinical indication and provided consent. Exclusion criteria: refusal of consent (none) and severely restricted mouth opening (none). |
|  |  | (c) Yes. Figure 2. STROBE flow diagram showing patient screening, enrolment, follow-up, and analysis. |
| Descriptive data | 14* | (a) Yes. Table 1. Age, gender, BMI, ASA, Mallampati, mouth opening, thyromental distance, jaw protrusion, SAS, AS. |
|  |  | (b) Yes. No missing data for any variable. Results, section 3. |
|  |  | (c) Yes. Methods, section 2.4. 24-hour postoperative follow-up; all patients remained hospitalized. |
| Outcome data | 15* | Yes. Results, Tables 2–4. Intubation time, success rate, haemodynamic parameters, adverse events reported. |
| Main results | 16 | (a) Yes. Results, Table 4. Repeated-measures ANOVA results with F-statistics, p-values, partial η², and 95% CIs for post-hoc comparisons. Confounders discussed narratively in Discussion 4.1. |
|  |  | (b) NA. Continuous variables (age, BMI, intubation time) were analysed as continuous; no categorisation applied. |
|  |  | (c)NA. Not applicable to this feasibility study. |
| Other analyses | 17 | NA. No subgroup or sensitivity analyses performed. |
| Discussion | | |
| Key results | 18 | Yes. Discussion, paragraph 2. Summarised primary findings and their relationship to feasibility objectives. |
| Limitations | 19 | Yes. Discussion, section 4.5. Limitations comprehensively discussed: sample size, single-centre, fixed operators, difficult-airway subgroup, ASA distribution, learning curve, zero-event CIs, uncontrolled design, statistical uncertainty (95% CI 91.2–100%). |
| Interpretation | 20 | Yes. Discussion, section 4.6. "Based on these preliminary feasibility data... findings are preliminary... warrants further evaluation... before routine adoption can be recommended." Also Conclusion. |
| Generalisability | 21 | Yes. Discussion, section 4.5. Limited generalisability due to single-centre design, fixed expert operators, ASA II-predominant cohort. |
| Other information | | |
| Funding | 22 | Yes. Conflicts of interest. "The authors declare that the research was conducted in the absence of any commercial or financial relationships..." No specific funding reported. |

*Give information separately for exposed and unexposed groups.

**Note:** An Explanation and Elaboration article discusses each checklist item and gives methodological background and published examples of transparent reporting. The STROBE checklist is best used in conjunction with this article (freely available on the Web sites of PLoS Medicine at http://www.plosmedicine.org/, Annals of Internal Medicine at http://www.annals.org/, and Epidemiology at http://www.epidem.com/). Information on the STROBE Initiative is available at http://www.strobe-statement.org.
